# Development of an ^123^I-metaiodobenzylguanidine Myocardial Three-Dimensional Quantification Method for the Diagnosis of Lewy Body Disease

**DOI:** 10.22038/aojnmb.2018.10595

**Published:** 2018

**Authors:** Yoshito Kamiya, Satoru Ota, Yuta Tanaka, Kosuke Yamashita, Akihiro Takaki, Shigeki Ito

**Affiliations:** 1 Graduate School of Health Sciences, Kumamoto University, Kumamoto, Japan; 2 Chibana Clinic, Okinawa, Japan; 3 Faculty of Life Sciences, Kumamoto University, Kumamoto, Japan; 4 Department of Medical Imaging, Faculty of Life Sciences, Kumamoto University, Kumamoto, Japan

**Keywords:** Iodine-123 metaiodobenzylguanidine, Lewy body disease, Quantification, Single-photon emission tomography

## Abstract

**Objective(s):**

We recently developed a new uptake index method for ^123^I-metaiodobenzylguanidine (^123^I-MIBG) heart uptake measurements by using planar images (radioisotope angiography and planar image) for the diagnosis of Lewy body disease (LBD), including Parkinson’s disease (PD) and dementia with Lewy bodies (DLB). However, the diagnostic accuracy of the uptake index was approximately equal to that of the heart-to-mediastinum ratio (H/M) for the discrimination of the LBD and non-LBD patients. A simple and pain-free uptake index method using ^123^I-MIBG SPECT images by modifying the uptake index method may show better diagnostic accuracy than the planar uptake index method. We hypothesized that the development of a new uptake index method for the determination of ^123^I-MIBG using single-photon emission computed tomography (SPECT) imaging would provide a reliable and reproducible diagnostic tool for clinical application. Regarding this, the purpose of this study was to develop a new uptake index method with a simple protocol to determine ^123^I-MIBG uptake on SPECT.

**Methods:**

The ^123^I-MIBG input function was determined from the input counts of the pulmonary artery, assessed by analyzing the pulmonary artery time-activity curves. The ^123^I-MIBG output function was obtained from ^123^I-MIBG SPECT counts on the polar map. The uptake index was calculated through dividing the output function by the input function (SPECT uptake method). For the purpose of the study, 77 patients underwent ^123^I-MIBG SPECT, with an average of 31.5 min after clinical assessment and injection of the tracer. The H/M values, as well as planar and SPECT uptake indices were calculated, and then correlated with clinical features.

**Results:**

According to the results, values obtained for LBD were significantly lower than those for non-LBD in all analyses (P<0.01). The overlap of the H/M values between the LBD and non-LBD cases ranged from 2.06 to 2.50. Furthermore, the overlap in uptake index values between LBD and non-LBD cases in planar image analysis was 1.05-1.29. The SPECT uptake index method showed the least overlap of 1.23-1.25, with the highest value for LBD patients clearly distinguished from the lowest value for the non-LBD patients.

**Conclusion:**

The new ^123^I-MIBG SPECT quantification method, developed by the input counts of the pulmonary artery, clearly distinguished LBD from non-LBD. Therefore, this method may be appropriate for routine clinical study.

## Introduction

Iodine-123 meta-iodobenzylguanidine (^123^I-MIBG) has been used to evaluate cardiac sympathetic denervation in Lewy body disease (LBD), including Parkinson’s disease (PD) and dementia with Lewy bodies (DLB) (1-3). However, the heart-to-mediastinum (H/M) ratios obtained by means of ^123^I-MIBG myocardial scintigraphy show limited accuracy due to the use of the low counts of the mediastinum as background counts (4-6). 

We recently developed a new uptake index method for ^123^I-MIBG heart uptake measurements by using two-dimensional (2D) images (radioisotope [RI] angiography and planar image) (7). This method does not depend on the system performance of the gamma cameras and requires only simple count analysis without an experimental count-conversion calculation (7). However, the diagnostic accuracy of this uptake index was approximately equal to that of the H/M for the discrimination of LBD and non-LBD patients (7). 

The^ 123^I-MIBG semi-quantification methods using single-photon emission tomography (SPECT) images, proposed by Chen et al. and van der Veen et al. (8, 9), depend on the system performance of the gamma cameras. The ^123^I-MIBG retention index was calculated by determining the ratio of the arterial-blood counts, and myocardial counts were obtained by the ^123^I-MIBG cadmium zinc telluride SPECT image analysis (10). However, these methods require complex procedures (8-10). Therefore, a simple procedure and pain-free (non-invasive) imaging procedure is required in order to guarantee repeatability and reproducibility for routine clinical study. 

If a simple and pain-free uptake index method using ^123^I-MIBG SPECT imaging can be developed by modifying the 2D uptake index method (7), the diagnostic accuracy of this method may be superior to that of the 2D uptake index method. We hypothesized that the development of a new uptake index method for the determination of ^123^I-MIBG using SPECT imaging would provide a reliable and reproducible diagnostic tool for clinical application. Therefore, the aim of this study was to develop a new uptake index method with a simple protocol to determine ^123^I-MIBG uptake on SPECT.

## Methods

***Theory***

**Figure 1:**
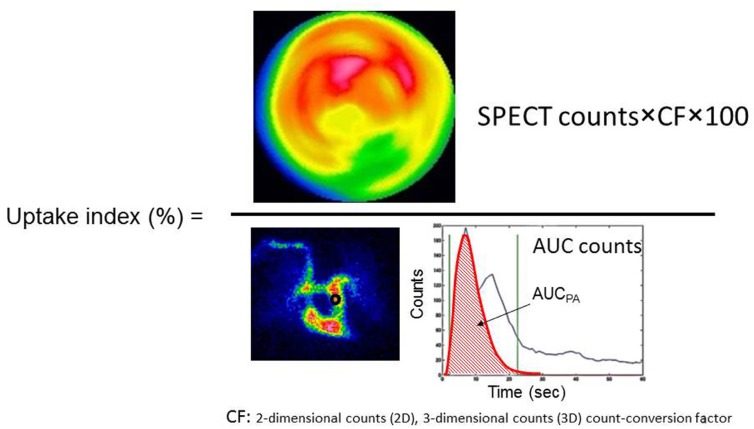
Region of interest (ROI) settings and typical time-activity curves of the pulmonary artery

Figure 1 shows the count time-activity curve (TAC) of the pulmonary artery on dynamic chest images. The first peak of the TAC was fitted with a gamma function, and the area under the curve (AUC) was obtained by integrating the gamma functions (Figure 1). The input count obtained using the integrated counts of the TAC on the pulmonary artery region of interest (ROI) of the chest RI angiography images is directly proportional to the administered dose (7). The uptake index can be noninvasively calculated by using the AUC, as follows:

(Eq. 1) $$\mathrm{Uptake index}=(C_{\mathrm{myo}}(t)\times {\mathrm{CCF}}_{\mathrm{SPECT}})/(\mathrm{AUC}\times {\mathrm{CCF}}_{\mathrm{planar}})$$

where, $C_{\mathrm{myo}}(t)$ (counts/mL) is the concentration of the radioactivity in the myocardium at the time of *t* after ^123^I-MIBG infusion.

The constants ${\mathrm{CCF}}_{\mathrm{SPECT}}$ and ${\mathrm{CCF}}_{\mathrm{planar}}$ are coefficients for converting the units from counts/mL to kBq/mL. These constants have been experimentally estimated by linear regression analysis. Dynamic images allow 2D counts, whereas SPECT images facilitate 3D counts. Therefore, a 2D-3D count-conversion factor (CF) between planar counts and SPECT counts is necessary. 

(Eq. 2) $$\mathrm{CF}={\mathrm{CCF}}_{\mathrm{SPECT}}/{\mathrm{CCF}}_{\mathrm{planar}}$$

The *CF* is specific to each facility, because it depends on the performance of SPECT scanner. Therefore, uptake index defined as:

(Eq. 3) $$\mathrm{Uptake index}=(C_{\mathrm{myo}}(t)\times \mathrm{CF})/(\mathrm{AUC})$$

The constants ${\mathrm{CCF}}_{\mathrm{SPECT}}$, CCFplanar and CF  have been estimated experimentally.

***Study protocol***

***Chest reconstructive increment angiography***

To determine the input function, ^123^I-MIBG dynamic planar images of the chest in the anterior view were obtained for a period of 2 min (1 s/frame, 128×128 matrix, 3.3 mm/pixel) using a detector equipped with low- and medium-energy general purpose collimators after a bolus injection of 111 MBq of ^123^I-MIBG.

A small amount of ^123^I-MIBG remains in the lungs and administration dose of ^123^I-MIBG is only 111 MBq (7, 11). Therefore, the ROI on the ascending aorta cannot be set on the chest RI angiography images due to the low activity level of ^123^I-MIBG. The TAC of the pulmonary artery was obtained by placing a circular ROI with a diameter of 3 pixels on the dynamic images of the pulmonary artery (7). The AUC of the first peak was fitted using the gamma function. The input count was obtained by using the integrated counts of the TAC on the pulmonary artery ROI of the chest RI angiography images (7, 12, 13). These processes were performed by using our original fully automatic program (14).

***Planar image***

**Figure 2:**
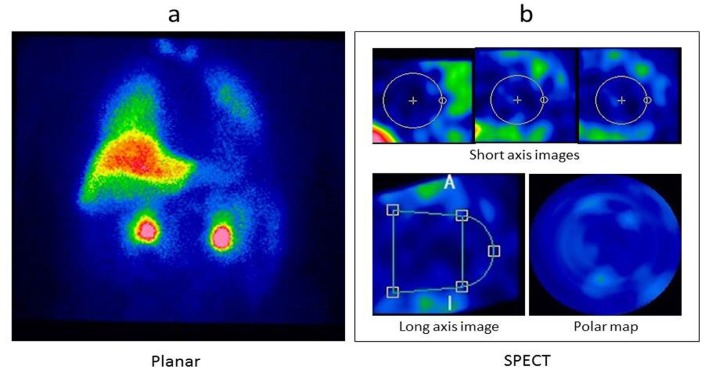
Region of interest setting and image reconstruction for the low ^123^I-MIBG uptake images

To determine the output function, a chest planar image was collected after dynamic image acquisition for 5 min (256×256 (zoom factor, 1.45; 3.3 mm/pixel)) and the conventional heart-to-mediastinum ratio and uptake on the planar image were recorded. For the planar image with low cardiac MIBG uptake, the ROI was set by virtually assuming a myocardium as depicted in Figure 2a.

^123^ ***I-MIBG SPECT***

The^ 123^I-MIBG SPECT imaging was performed using dual-head SPECT scanners (E-cam, Toshiba, Japan, collimator: LMEGP) in order to calculate the uptake on the SPECT images. The SPECT was performed with the average scan time of 31.5 min using a scanner equipped with an LMEGP collimator. Projection data were acquired every 20 sec by continuously rotating the detector by 360° (60 steps/360°/20 sec, 64×64 matrix, zoom factor of 1.45). 

The SPECT images were obtained using the ordered subsets expectation maximization method (subsets 4, iterations 30). Scatter and attenuation corrections were not performed on the SPECT images. For the SPECT images with low cardiac MIBG uptake, the short-axis myocardial images for the polar map were reconstructed by assuming a myocardium virtually as illustrated in Figure 2b.

The output function was obtained by using the ^123^I-MIBG SPECT counts on the polar map. The uptake was obtained by dividing the output function by the input function. For the purpose of the study, 77 patients underwent ^123^I-MIBG SPECT, 25 min after the tracer injection and clinical features had been assessed, including Hoehn and Yahr staging (15). 

The conventional H/M was calculated using the method reported by Okuda et al. (16). The same heart ROI was used for the calculation of conventional H/M and planar uptake index (7). The correlations between the H/M, planar uptake, and SPECT uptake were analyzed with respect to the observed clinical features.

***Subjects***

Images collected from 77 patients with the mean age of 74.7 years (37 men and 40 women, age range: 56-92 years) were used to develop the theory and procedure of this new quantification method (Table 1). The patients underwent both ^123^I-MIBG chest reconstructive increment angiography and SPECT examinations (^123^I-MIBG examinations) within January 2014 to August 2015 at Chibana Clinic, Okinawa, Japan. As listed in Table 2, the study population consisted of 77 patients, including 27 patients with idiopathic Parkinson’s disease, 10 patients with dementia with Lewy bodies, 8 patients with Alzheimer-type disease with Parkinsonism, 13 patients with vascular Parkinsonism, and 9 patients with drug-induced Parkinsonism. In addition, 10 patients had other conditions, such as unclassifiable Parkinsonism (n=5), depression (n=2), mild cognitive impairment (n=2), anxiety neurosis (n=1), and gait disturbance (n=1). 

None of the patients had pulmonary disease. The study was approved by the Ethics Committee of Medicine at Chibana Clinic and the Kumamoto University for Human Studies. Written informed consent was obtained from all patients prior to the study. All image data were handled anonymously.

***Statistical analysis***

Fisher’s exact test was employed to evaluate the differences between the binary variables with respect to gender. Continuous variables were presented as mean, standard deviation, and median. Where indicated, differences were determined using a two-sided Student’s t-test or Mann-Whitney U test. The Spearman rank correlation was calculated to assess the correlation between the two studies. Data analysis was performed in MedCalc software (version 12.4). 

The diagnostic values of the H/M, planar uptake, and SPECT uptake methods were assessed by calculating the area under the receiver operating characteristic (ROC) curve. Diagnostic accuracy was evaluated by calculating sensitivity, specificity, positive predictive value (PPV), and negative predictive value (NPV). The value criterion for each method was based on the Youden index of the ROC curve. 

These analyses were performed in ROCKIT software developed by the University of Chicago. In this software, the quasi-maximum likelihood estimation of the binormal distribution was fitted to the uptake indices (17). The Dorfman-Berbaum-Metz method was run to compare the AUC values between the two methods (18). P-value less than 0.05 was considered statistically significant. 

## Results

**Figure 3.**
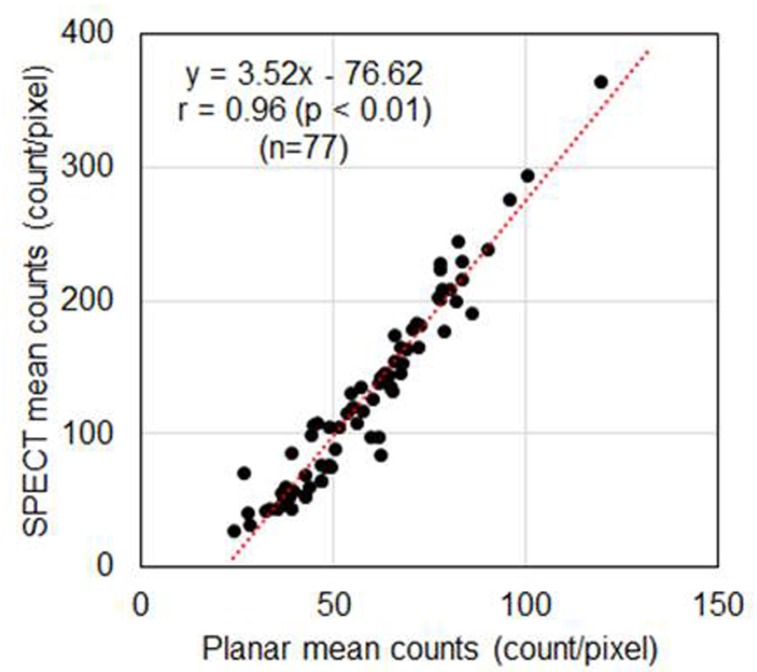
Correlation between the mean planar mean counts and SPECT counts

We estimated the correlation between the mean myocardial counts obtained based on the polar map of the SPECT images (SPECT counts) and the mean myocardial counts of the planar image (planar counts). The results revealed a significant linear correlation between the SPECT counts and planar counts (r=0.96, P<0.01, n=77). The conversion of the planar counts using the planar to SPECT count conversion coefficient yielded similar values to the SPECT counts (Figure 3).

**Figure 4:**
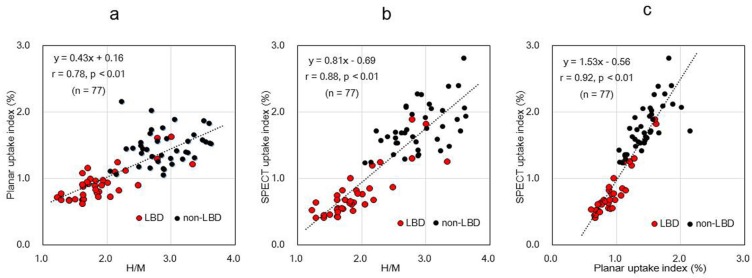
Correlation between heart-to-mediastinum ratio and uptake index percentage

The correlations among the conventional H/M, planar, and SPECT uptake index are depicted in Figure 4. Significant correlations were observed between the H/M and planar uptake values (r=0.78, P<0.01, n=77) (Figure 4a) and between H/Ms and SPECT uptake (r=0.88, P<0.01, n=77) (Figure 4b). Furthermore, there was a significant correlation between the planar uptake and SPECT uptake values (r=0.92, P<0.01, n=77) (Figure 4c).

**Figure 5:**
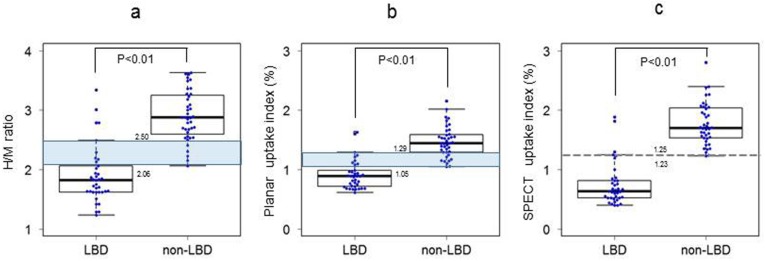
Comparison of the Lewy body disease and non-Lewy body disease groups in terms of the values obtained from each method

The comparisons of H/M values from the LBD and non-LBD patients, determined by conventional methods, displayed in Figure 5a. The H/Ms for the LBD patients were lower than those for the non-LBD patients (P<0.01). The H/Ms of the LBD patients ranged within 1.23 to 3.34 (median: 1.82, interquartile range [IQR]: 1.62-2.06, variance: 0.228, 95% confidence interval [CI]: 1.74-2.06) and those of the non-LBD patients ranged from 2.06 to 3.63 (median: 2.88, IQR: 2.61-3.24, variance: 0.178, 95% CI: 2.77-3.04). The highest H/M of the LBD patients (2.06) overlapped with the lowest H/M of the non-LBD patients (2.50).

The comparison of the uptake values obtained for the LBD and non-LBD patients using the planar uptake methods is displayed in Figure 5b. The values for the LBD patients were lower than those for the non-LBD patients (P<0.01). The uptake values of the LBD patients ranged 0.61-1.63 (median: 0.89, IQR: 0.72-0.99, variance: 0.061, 95% CI: 0.83-0.99), and those of the non-LBD were within 1.05-2.16 (median: 1.44, variance: 0.068, 95% CI: 1.38-1.55). The overlap between the uptake values of the LBD and non-LBD patients occurred between 1.29 and 1.05.

Figure 5c presents the comparison of the uptake values of the LBD and non-LBD patients using the SPECT uptake method. The values for the LBD patients were significantly lower than those for the non-LBD patients (P<0.01). The uptake values for the LBD patients ranged between 0.40 and 1.89 (median: 0.63, IQR: 0.52-0.82, variance: 0.130, 95% CI: 0.63-0.87) and those for the non-LBD patients were between 1.23 and 2.80 (median: 1.70, IQR: 1.53-2.03, variance: 0.127, 95% CI: 1.65-1.88). The highest value obtained from the LBD patients was clearly distinguished from the lowest value obtained from the non-LBD patients.

**Figure 6:**
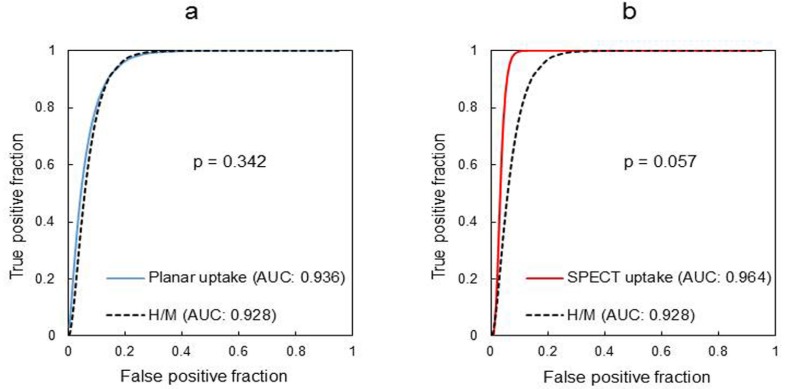
Receiver-operating characteristic (ROC) curves

Figure 6 presents the ROC curves of the H/M, planar uptake index, and SPECT uptake index methods. The AUC values of SPECT uptake index and the planar uptake index were 0.96 and 0.93, respectively. The AUC of the planar uptake index method (AUC: 0.93) was approximately equal to that of the H/M method (AUC: 0.94, P=0.34) (Figure 6a). Moreover, the SPECT uptake index had a higher AUC value than that of the H/M method (Figure 6b). The SPECT uptake index method and the H/M method showed a statistically significant difference in terms of the ROC curves (P=0.057) (Figure 5b). 

Table 3 compares the diagnostic accuracy of the H/M, planar, and SPECT uptake index methods. The SPECT uptake index method (AUC: 0.96, sensitivity: 0.86, specificity: 1.00, PPV: 1.00, NPV: 0.89, accuracy: 0.94) yielded higher diagnostic performance for non-Lewy body disease, compared to the H/M (AUC: 0.93, sensitivity: 0.84, specificity: 0.95, PPV: 0.94, NPV: 0.86, accuracy: 0.90) and planar uptake index methods (AUC: 0.94, sensitivity: 0.81, specificity: 0.93, PPV: 0.91, NPV: 0.84, accuracy: 0.87). 

## Discussion

In the present study, we established a new quantification method for myocardial ^123^I-MIBG uptake measurements by analyzing the chest dynamic planar and SPECT images. The ^123^I-MIBG input function was calculated by modifying the simple non-invasive ^123^I-IMP quantification based on a microsphere model according to the pharmacokinetics of ^123^I-MIBG (7, 12-14, 19).

**Table 1:** Characteristics of the subjects

**Classification**	**LBD group (n=37)**	**non-LBD group (n=40)**	***p*** ***-value*** *
Sex (M/F)	17/20	20/20	0.821
Age (y)	75.0±7.6 (range 57-89)	74.4±7.7 (range 56-92)	0.934

* Fisher’s exact test.

**Table 2:** Clinical diagnoses of the subjects

**LBD group **		**non-LBD group**	
PD	27	AD	8
DLB	10	VPS	13
		DPS	9
		Other	10
Total	37		40

LBD: Lewy body disease

DLB: Dementia with Lewy bodies

PD: Parkinson’s disease

AD: AD with parkinsonism

VPS: Vascular parkinsonism

DPS: Drug-induced parkinsonism

**Table 3.** Comparison of diagnostic accuracy (n=77)

**Method**	**AUC**	**Sensitivity**	**Specificity**	**Criterion values**	**95% CI**	**PPV** *	**NPV** †	**Accuracy**
H/M	0.93	0.84	0.95	<2.21	0.84 - 0.97	0.94	0.86	0.90
Planar uptake index	0.94	0.81	0.93	<1.1	0.85 - 0.98	0.91	0.84	0.87
SPECT uptake index	0.96	0.86	1.00	<1.2	0.86 - 0.99	1.00	0.89	0.94

AUC, area under the curve; 95% CI, 95% confidence interval.

H/M, heart to mediastinum ratio.

* PPV, positive predictive value;

† NPV, negative predictive value.

Typical time-activity curves of the pulmonary artery

The first peak of the time-activity curve was fitted with a gamma function, and the area under the count-time-activity curve was obtained by integrating the gamma functions

a: For the planar image, the region of interest was set by assuming a myocardium virtually

b: For SPECT, the short-axis myocardial images for the polar map were reconstructed by assuming a myocardium virtually

No.27 (PD); H/M: 1.63, SPECT uptake index: 0.42%

a: Correlation between the conventional heart-to-mediastinum ratio and uptake index percentage using planar image analysis

b: Correlation between the conventional heart-to-mediastinum ratio and uptake index percentage using SPECT analysis

c: Correlation between planar uptake and SPECT uptake percentage

a: Comparison of the Lewy body disease and non-Lewy body disease groups based on the heart-to-mediastinum ratios

b: Comparison of the uptake index in the Lewy body disease and non-Lewy body disease groups based on planar image analysis

c: Comparison of the uptake index in the Lewy body disease and non-Lewy body disease groups based on SPECT analysis

a: ROC curves of the heart-to-mediastinum ratio and planar uptake index

b: ROC curves of the heart-to-mediastinum ratio and SPECT uptake index

The output function is determined from the total counts of the polar map of the myocardium. This is indicated by the maximum number of counts within each sector and calculated as the maximum average counts along three consecutive pixels (20). Therefore, the total counts from the polar map are different from the absolute myocardial counts.

A very good linear correlation was shown between the planar and SPECT uptake indices (Figure 4c). Accordingly, it can be concluded that the myocardial count on the planar image is directly proportional to the total counts of the polar map using the myocardial SPECT images. This was due to obtaining uptake indices of the planar and SPECT images from the same input function. Therefore, the total counts of the myocardial SPECT polar map can be used to calculate the uptake index. 

The use of SPECT MIBG analysis methods has been previously proposed; however, the complexity of the required procedure meant that the reproducibility of the ROI that was used to define the SPECT method was not reliable (8, 9). The proposed SPECT uptake index method, which uses the total counts of the polar map, will improve the reproducibility of this method. 

We showed that the LBD and non-LBD patients can be distinguished using the H/M, as well as planar and SPECT uptake index methods (Figure 5, Table 3). In both H/M and planar uptake index methods, the highest values of the LBD patients overlapped with the lowest values of the non-LBD patients (Figures 5a, b). These results were approximately equal to those obtained in our previous report (7). 

In contrast, the highest SPECT uptake index values of the LBD patients were clearly distinguished from the lowest values of the non-LBD patients (Figure 5c). The diagnostic performance of the planar uptake index method may be approximately equal to that of the H/M method (Figure 6a, Table 3). On the contrary, SPECT uptake index had a higher AUC value than that of the H/M method and the result of statistical test showed a tendency to be statistically significant (Figure 6b).

Furthermore, this method is an improved version of the conventional H/M and planar uptake index methods. Neither the planar uptake nor SPECT uptake methods depend on the system performance of the gamma cameras due to activity calculation (kBq/mL). Therefore, the SPECT uptake index method can be applied in the routine clinical practice. 

For SPECT images with low cardiac MIBG uptake, the short-axis myocardial images for the polar map were reconstructed by virtually assuming a myocardium. Consequently, the SPECT uptake index should not be used for low cardiac MIBG uptake patients. These patients can be clarified by using the planar uptake index method before SPECT imaging. 

The use of the planar uptake and SPECT uptake index is entirely suitable for routine clinical studies. When the values obtained by means of the planar uptake index method are within the overlap range, it is recommended to use the SPECT uptake index method to enable distinction between the LBD and non-LBD patients. Moreover, the SPECT uptake index method will simplify and increase examination usefulness.

The SPECT image analysis requires scatter and attenuation correction, as well as resolution recovery (21-23). However, we did not perform attenuation correction for the SPECT images collected during this study. Nevertheless, the LBD and non-LBD patients were clearly differentiated on the basis of the non-attenuation corrected SPECT images, as shown in Figure 5c. 

The difference between the LBD and non-LBD patients regarding the uptake values derived from the fact that the attenuation-corrected SPECT images was probably greater than that of the non-attenuation-corrected SPECT images. Accordingly, we expected that non-attenuation-corrected SPECT images may be applied in clinical study.

This simple protocol for using the planar and SPECT uptake index method with ^123^I-MIBG SPECT uptake measurements will be easily transferrable for the evaluation of the cardiac sympathetic nerve function. The administration dose is obtained by analyzing the dynamic planar images of the chest as an index of the input function in the planar uptake and SPECT uptake index methods. 

The development of an automatic algorithm that can define the ROI for the determination of the input function in this method facilitates the completion of the subsequent analysis of the uptake index within a few minutes through a fully automated uptake analysis program, without the need for complex techniques. This automated program would not only improve the repeatability and reproducibility of this uptake method, but also be available for the non-invasive quantification of other examinations within the ambit of nuclear medicine.

The results of this study were obtained from the patients accessing one facility. Further studies are needed to confirm the feasibility of this method across multiple facilities. In addition, the validity and accuracy of this method would ideally need to be confirmed by SPECT in a different patient group.

## Conclusion

This new quantification method was developed using ^123^I-MIBG SPECT and the input counts of the pulmonary artery. This method clearly distinguishes between the LBD and non-LBD patients and could be applied in the clinic setting for routine study. Further testing is necessary to confirm the feasibility of this method in other facilities.

## Disclosure

This study was supported by grants (Grant-in-Aid for Scientific Research (C) 26461860) obtained from the Ministry of Education, Culture, Sports, Science, and Technology of Japan. No other potential conflict of interest relevant to this article was reported.
